# Treatment of Maxillary Deficiency by Miniplates: A Case Report

**DOI:** 10.5402/2011/854924

**Published:** 2011-05-10

**Authors:** Rahman Showkatbakhsh, Abdolreza Jamilian, Mohammad Behnaz

**Affiliations:** ^1^Department of Orthodontics, Shahid Beheshti University, Tehran 19468 53314, Iran; ^2^Department of Orthodontics, School of Dentistry, Islamic Azad University, Tehran 19668 43133, Iran

## Abstract

*Introduction*. Numerous devices have been introduced for correction of Class III malocclusion and maxillary deficiency. *Aim*. To assess the dentoskeletal effects of miniplates combined with Class III traction in treating Cl III malocclusion and maxillary deficiency in growing patients. *Methods*. This case describes the treatment of a maxillary-deficient 11-year-old boy by using miniplates. The patient's parents rejected the use of extraoral appliances and major surgical correction; therefore the treatment was done by using Class III elastics connected from two mandibular miniplates to an upper removable appliance. Two miniplates were inserted in the anterior part of the mandible in the canine areas under local anaesthesia. The treatment lasted for 10 months after which favourable correction of the malocclusion was observed. *Results*. The SNA and ANB angles increased by 5.1° and 4.4°, respectively. Lower 1 to mandibular plane decreased by 3.4°. *Conclusions*. This case demonstrates that miniplates can be a suitable method to extraoral appliances and major surgery in maxillary deficiency cases.

## 1. Introduction

Skeletal Class III malocclusion is one of the most difficult discrepancies to correct. Skeletal Class III anomalies are associated with maxillary retrusion, mandibular protrusion, or both [1, 2]. It has been found that 65% to 67% of all Class III malocclusions were characterized by maxillary deficiency [3]. In subjects with maxillary deficiency where the mandible is not markedly affected, treatment may involve stimulation and guidance of maxillary growth by orthopaedic forces. Various types of extraoral appliances, such as facemasks and reverse pull headgears have been used to correct maxillary deficiency; [4–6] however, there are problems with patient compliance due to their size and appearance.

Dental implants, miniplates, and modified fixation screws provide bone anchorage in orthodontic treatment [7–9]. Miniscrews (mini-implants) have also become popular because they are easier to both insert and remove [10, 11].

In this case report, two miniplates were inserted in the anterior part of the mandible in the canine areas and connected to a removable appliance in the upper jaw by use of elastics in order to correct maxillary deficiency.

## 2. Diagnosis and Etiology

The patient was an 11-year-old boy who was referred for treatment of maxillary deficiency. He had no medical problems, and there were no signs of temporomandibular joint dysfunction. The patient had a skeletal Class III malocclusion and maxillary deficiency. His parents had no Class III characteristics.

**Figure 1:**
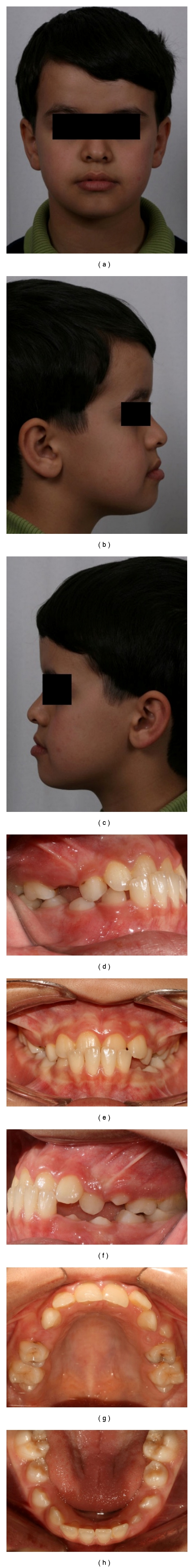
Pretreatment photos of the patient.

**Figure 2:**
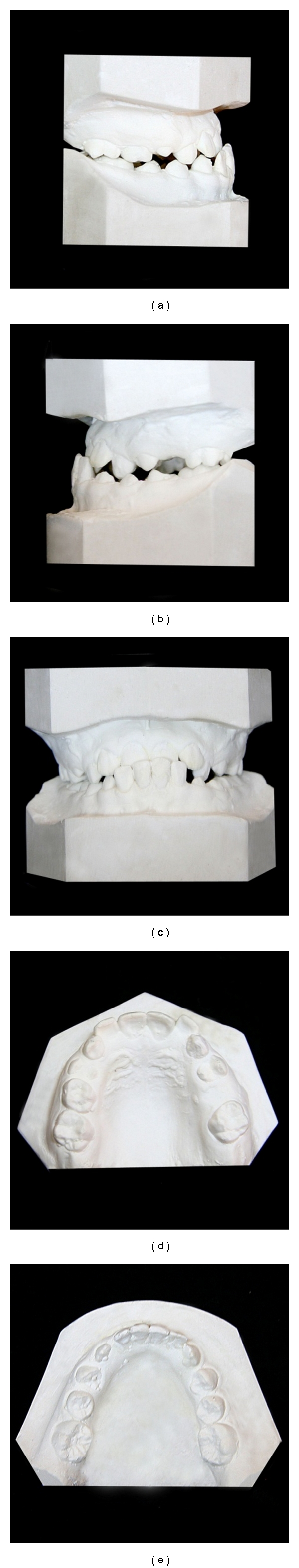
Pretreatment photos of the dental casts.

**Figure 3:**
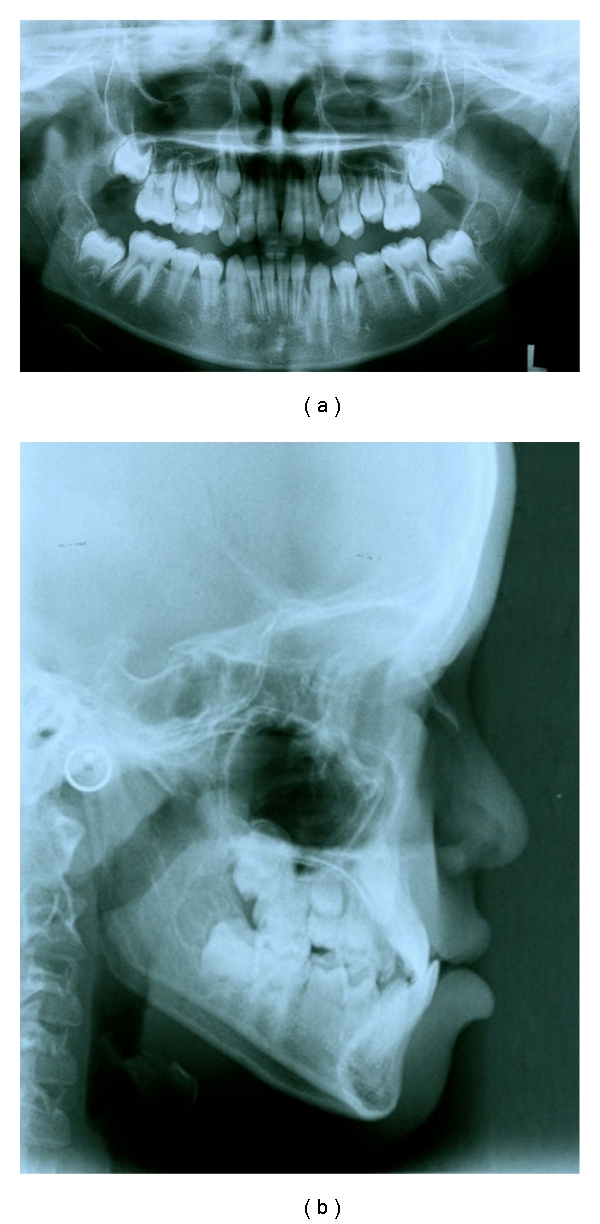
Pretreatment OPG and lateral cephalogram of the patient.

The facial photographs showed a Class III appearance with a concave profile because of maxillary deficiency. The pretreatment intraoral photographs and dental casts showed Class III relationship of the central incisors and anterior crossbite. The patient had a Class III molar relationship on the right and Class I on the left side (Figures 1 and 2). Cephalometric analysis confirmed the Class III skeletal pattern (Table 1) (Figure 3).

## 3. Treatment Objectives

The treatment objectives for this patient were to

1. correct the deficient maxillary arch, ideally by forward positioning of the maxilla;
2. obtain an ideal overjet and overbite;
3. correct the anterior crossbites.

## 4. Treatment Alternatives

 Extraoral appliances, such as protraction facemask, Class III functional appliance, any modified maxillary protraction devices, and orthognathic surgery, were considered as alternative treatments for the correction of this Class III malocclusion. However, the patient refused the use of extraoral appliances and major surgery. Therefore, in this case, it was decided to use miniplates to protract the maxilla by application of Class III elastics.

## 5. Treatment Progress

Plates for Orthodontic Anchorage (Junji Sugawara, D.D.S., Ph.D.) (AP-YL-013) were placed under local anaesthesia in the canine areas of the mandible by a maxillofacial surgeon. The ideal position for miniplates insertion was evaluated by using a panoramic radiograph in order to avoid damage to the roots of the adjacent teeth and mental foramen. A tightly fitting and well-retained upper removable appliance was fabricated with two Adams clasps on the upper first permanent molars. Each of the Adams clasps had a loop which was used for retaining the elastics. A labial bow was also used on the anterior teeth for retention. A maxillary posterior bite plate was used to disclude the upper and lower jaws. 

**Figure 4:**
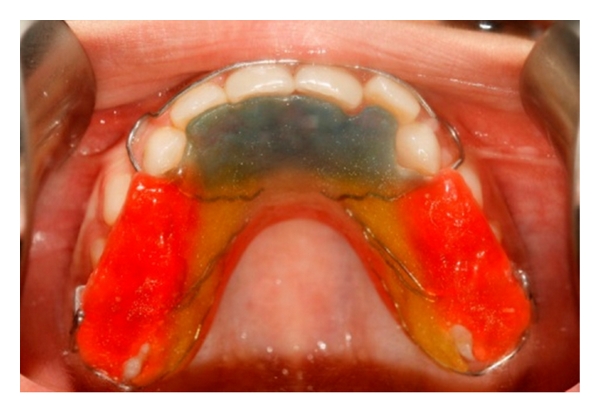
Removable appliance in the upper jaw.

Orthodontic latex elastics (3/16′′ heavy size—Unitek Elastics) were connected from the hooks of the miniplates to the Adams clasps of the removable appliance to generate approximately 500 g of anterior retraction. The patient was instructed to wear the appliance full-time except for eating, contact sports, and tooth brushing; he was also told to change the elastics every day. In order to retain these elastics, the Adams clasps on the molars were bent to form loops (Figure 4).

## 6. Treatment Results

**Figure 5:**
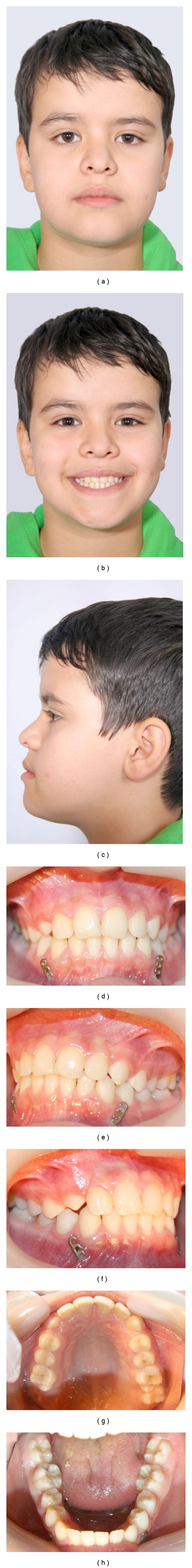
Posttreatment photos of the patient.

**Figure 6:**
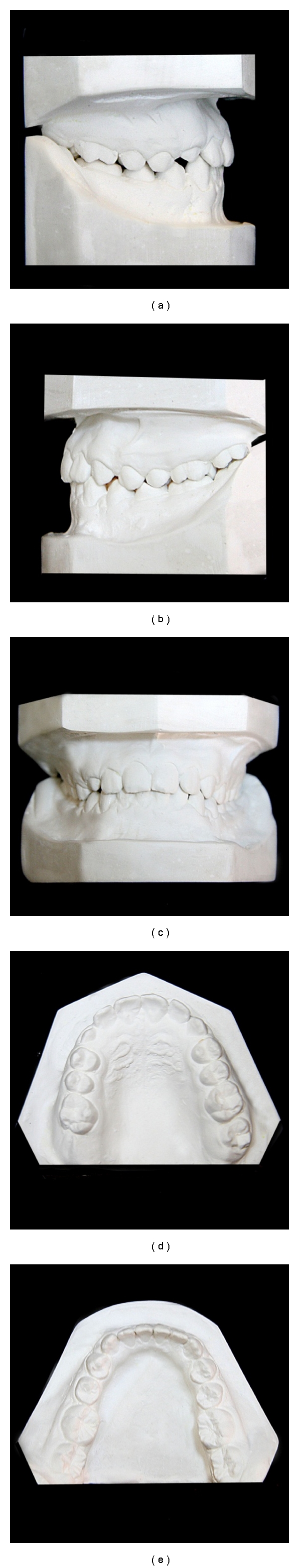
Posttreatment photos of the dental casts.

**Figure 7:**
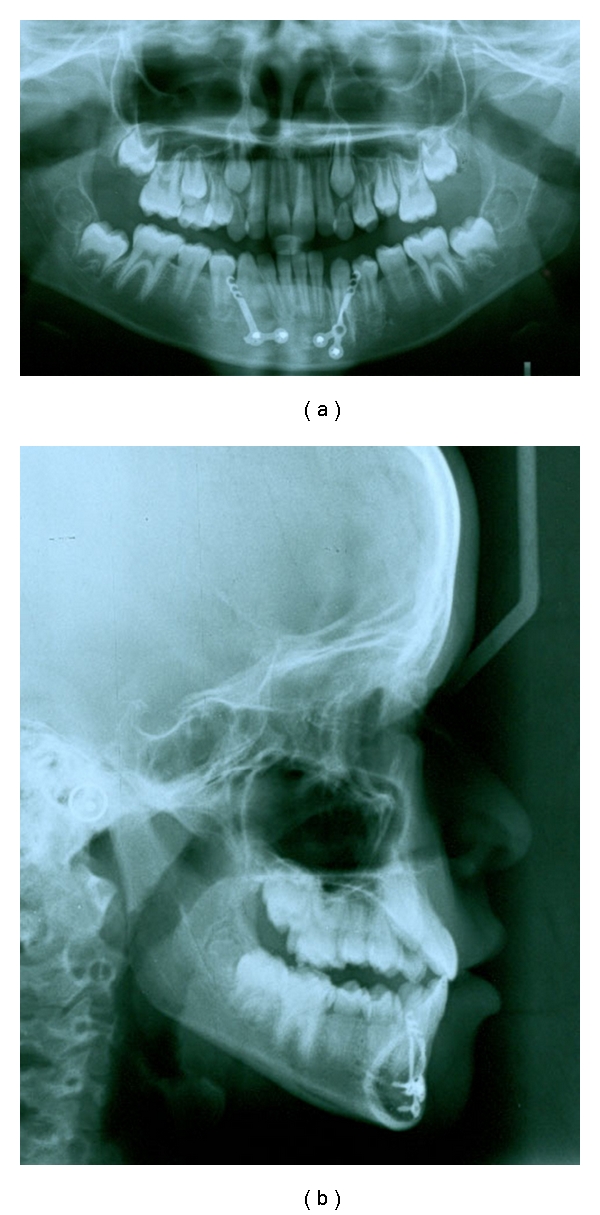
Posttreatment OPG and lateral cephalogram of the patient.

**Figure 8:**
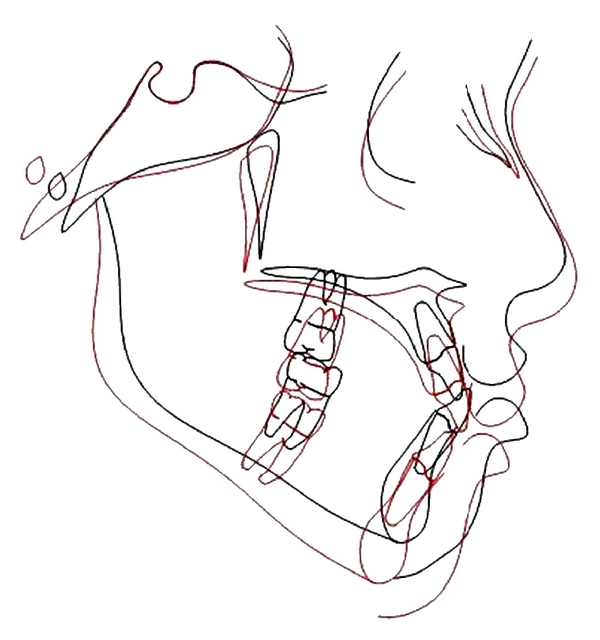
Superimposition in anterior cranial base at sella. Red: after treatment, black: before treatment.

After 10 months of active treatment a positive overjet and Class I buccal segments were achieved and the anterior crossbite was corrected (Figures 5 and 6). The posttreatment cephalometric radiograph tracing showed a favourable increase of 5.1° and 4.4° in the SNA and ANB angles, respectively, (Figure 7). The pre- and posttreatment cephalometric superimposition on the anterior cranial base is shown in Figure 8.

## 7. Discussion

This case demonstrates the clinical application of miniplates in the treatment of an 11-year-old boy with maxillary deficiency. Our system of treatment differs from conventional force applications, such as facemasks [4–6].

Previous studies [2, 12–20] show that a significant amount of maxillary forward movement can be produced with maxillary protraction appliances. Recent reports indicate that some anteroposterior changes can be achieved up to the beginning of adolescence; [6] however, these appliances may cause great discomfort for patients and are highly visible to wear, which leads to reduced patient cooperation. Another problem caused by extraoral appliances is that they can cause skin abrasions on the chin especially in hot climates. Therefore patients may simply refrain from wearing the appliance, and the lack of cooperation might lead to an unsatisfactory result.

One of the disadvantages of extraoral appliances is that, when extraoral force is applied against the chin, it is difficult to avoid tipping the lower incisors lingually. In other words, use of a chin cup can lead to lingual tipping of the lower incisors as a result of the pressure of the chin cup component on the lower lip and dentition [21]. In most cases, lingual tipping is an undesirable side effect and can cause crowding [22]. In a case report miniscrews [23] have been used for treatment of maxillary deficiency. One of the limitations of miniscrew is their loosening, which can be distressing for the clinician and the patient. In order to overcome this problem^,^ wider diameter and deeper insertion of miniscrews must be used. De Clerck et al. [24] used the miniplates to protract the maxilla however; the design of current case report is different from that study. In a recent study bone-anchored maxillary protraction (BAMP) with miniplates was used in patients with Class III malocclusion, and significant improvements of over jet and molar relationship were recorded [25].

In this case report, minor surgery and miniplates were used to overcome these various problems. As undertaken in this case, applying a force to the teeth in order to correct the skeletal discrepancy will inevitably result in tooth movement; [6] therefore, a full coverage upper removable appliance was used to cover all the maxillary dentition. The treatment process lasted for 10 months. However, since the patient was only 11 years old and still had considerable residual growth, treatment was continued by fixed appliance. 

The forces generated by elastics may be divided into two components. One force component is in a horizontal direction, moving the maxilla forwards, which is favourable in maxillary deficiency cases. The second component is in a vertical direction, moving the posterior maxillary dentition downwards. This might lead to unfavourable tooth movements in high angle cases, but it is not a problem in patients with a low or average face height. Maxillary posterior bite plate can overcome this problem in high angle cases by decreasing facial height.

## 8. Conclusions

This case report demonstrates a different method of using miniplates to treat an 11-year-old boy with a skeletal Class III malocclusion and maxillary deficiency. This treatment was found to be an acceptable alternative to the use of extraoral appliances such as facemasks and major surgery.

## Figures and Tables

**Table 1:** Cephalometric analysis at pretreatment, posttreatment.

	Pretreatment	Posttreatment
SNA	77.1°	82.2°
SNB	79.9°	80.6°
ANB	−2.8°	1.6°
GO-GN to SN	27°	34°
U1 to Sn	116°	110°
MMPA	27.6°	32°
Facial proportion	64.3%	58%
SN to MxPl	8.7°	5.5°
U1 to MxPl	115°	112.9°
L1 to MnPl	90°	90°
Interincisal angle	127°	128.5°
L1 to A-Pog line	9 mm	5.2 mm
